# Metabolically-inactive glucagon-like peptide-1(9–36)amide confers selective protective actions against post-myocardial infarction remodelling

**DOI:** 10.1186/s12933-016-0386-5

**Published:** 2016-04-14

**Authors:** Emma Robinson, Mitchel Tate, Samuel Lockhart, Claire McPeake, Karla M. O’Neill, Kevin S. Edgar, Danielle Calderwood, Brian D. Green, Barbara J. McDermott, David J. Grieve

**Affiliations:** Wellcome-Wolfson Institute for Experimental Medicine, Queen’s University Belfast, 97 Lisburn Road, Belfast, BT9 7AE UK; School of Biological Sciences, Institute for Global Food Security, Queen’s University Belfast, Belfast, BT9 5HN UK

**Keywords:** Glucagon-like peptide-1 (GLP-1), GLP-1(9–36)amide, Myocardial infarction, Cardiac remodelling

## Abstract

**Background:**

Glucagon-like peptide-1 (GLP-1) therapies are routinely used for glycaemic control in diabetes and their emerging cardiovascular actions have been a major recent research focus. In addition to GLP-1 receptor activation, the metabolically-inactive breakdown product, GLP-1(9–36)amide, also appears to exert notable cardiovascular effects, including protection against acute cardiac ischaemia. Here, we specifically studied the influence of GLP-1(9–36)amide on chronic post-myocardial infarction (MI) remodelling, which is a major driver of heart failure progression.

**Methods:**

Adult female C57BL/6 J mice were subjected to permanent coronary artery ligation or sham surgery prior to continuous infusion with GLP-1(9–36)amide or vehicle control for 4 weeks.

**Results:**

Infarct size was similar between groups with no effect of GLP-1(9–36)amide on MI-induced cardiac hypertrophy, although modest reduction of in vitro phenylephrine-induced H9c2 cardiomyoblast hypertrophy was observed. Whilst echocardiographic systolic dysfunction post-MI remained unchanged, diastolic dysfunction (decreased mitral valve E/A ratio, increased E wave deceleration rate) was improved by GLP-1(9–36)amide treatment. This was associated with modulation of genes related to extracellular matrix turnover (MMP-2, MMP-9, TIMP-2), although interstitial fibrosis and pro-fibrotic gene expression were unaltered by GLP-1(9–36)amide. Cardiac macrophage infiltration was also reduced by GLP-1(9–36)amide together with pro-inflammatory cytokine expression (IL-1β, IL-6, MCP-1), whilst in vitro studies using RAW264.7 macrophages revealed global potentiation of basal pro-inflammatory and tissue protective cytokines (e.g. IL-1β, TNF-α, IL-10, Fizz1) in the presence of GLP-1(9–36)amide versus exendin-4.

**Conclusions:**

These data suggest that GLP-1(9–36)amide confers selective protection against post-MI remodelling via preferential preservation of diastolic function, most likely due to modulation of infiltrating macrophages, indicating that this often overlooked GLP-1 breakdown product may exert significant actions in this setting which should be considered in the context of GLP-1 therapy in patients with cardiovascular disease.

## Background

It is well established that glucagon-like peptide-1 (GLP-1), which is now routinely exploited in the clinical management of hyperglycaemia associated with type 2 diabetes (e.g. exenatide, saxagliptin), exerts important cardiovascular actions in both health and disease [1]. For example, GLP-1 mediates key physiological effects on blood pressure, vascular tone, and cardiac structure and function [2–6], whilst it is widely reported to be protective against both experimental and clinical cardiovascular disease in normoglycaemia and diabetes [7–13]. With regard to potential cardiovascular applications of GLP-1 therapy, one of the most studied areas has been in the setting of ischaemic heart disease, to which diabetic patients are particularly predisposed [14]. It has been known for several years that both GLP-1 receptor (GLP-1R) agonists and dipeptidyl peptidase-4 (DPP-4) inhibitors, which increase endogenous GLP-1, protect cardiomyocytes from acute ischaemic damage and promote functional recovery after experimental myocardial infarction (MI) [15]. More recently, GLP-1 analogues have been reported to improve post-MI survival and cardiac structure/function in rodent models independently of infarct size and metabolic effects [16, 17], suggesting direct protection against ischaemic heart failure. Indeed, our group have built on these findings to show that the GLP-1 mimetic, exendin-4, exerts specific benefits on post-MI remodelling in normoglycaemia via selective actions on inflammation and the extracellular matrix (ECM), which are characteristic features of the diabetic heart [18, 19], highlighting cell-targeted GLP-1-based therapies as a potential novel approach in this setting.

Interestingly, whilst the cardiovascular effects of native GLP-1(7–36)amide and its stable analogues are well established, its breakdown product, GLP-1(9–36)amide [hereafter referred to as GLP-1(9–36)], which is produced further to degradation by DPP-4 and is metabolically inactive [20], also appears to mediate important signalling actions in the cardiovascular system. For example, GLP-1(9–36) is reported to promote dose-dependent vascular relaxation ex vivo and to inhibit hyperglycaemia-induced mitochondrial reactive oxygen species (ROS) generation both in vitro and in vivo [4, 5, 21, 22]. With regard to cardiac ischaemia, protective actions of GLP-1(7–36)amide and exendin-4 against reperfusion injury were found to be resistant to the GLP-1R antagonist, exendin(9–39), and to persist in GLP-1R knockout mice, with the former being inhibited by the DPP-4 inhibitor, sitagliptin, indicating that they were likely to be mediated by GLP-1(9–36) and/or GLP-1R-independent pathways [5, 23]. Indeed, acute treatment of ex vivo mouse hearts with GLP-1(9–36) prior to ischaemia–reperfusion resulted in improved functional recovery and reduced infarct size, and conferred additional cardioprotective actions to those mediated by exendin-4 [24], suggesting that the metabolically-inactive GLP-1 peptide may activate distinct cardiovascular signalling pathways. Notably, the evident importance of GLP-1(9–36)-mediated actions (together with the known pleiotropic actions of GLP-1 signalling) may at least partly explain the apparently disappointing results of recent large-scale DPP-4 inhibitor trials which failed to demonstrate any significant benefits on cardiovascular outcomes in diabetic patients [1, 25, 26]. It is therefore clearly important to assess the potential of cardioactive metabolite pathways to the reported protective actions of GLP-1. Further to our recent observation that GLP-1 mediates distinct beneficial effects on post-infarction remodelling, the aim of this study was to specifically investigate whether metabolically-inactive GLP-1(9–36) also plays a key role in this setting, by employing a normoglycaemic experimental model to directly assess its cardioprotective contribution. Here, we report selective GLP-1(9–36)-mediated protection against diastolic dysfunction and myocardial inflammation post-MI together with specific modulation of macrophage response genes, actions which are clearly important to consider when assessing the likely effectiveness of GLP-1 targeting strategies in this setting.

## Methods

### Experimental model

Female C57BL/6 J mice (8–12 weeks; Harlan UK) were used throughout this study and were housed under constant climatic conditions with free access to food and water. All experimental procedures were performed in accordance with the Guidance on the Operation of the Animals (Scientific Procedures) Act, 1986 (UK) and approved by the Queen’s University Belfast Animal Welfare and Ethical Review Body. Mice were randomised prior to permanent ligation of the left anterior descending coronary artery under 2 % isofluorane/oxygen anaesthesia (with pre/post-operative buprenorphine analgesia, 0.05 mg/kg i.m., as required) or sham surgery, which involved an identical procedure with the exception of coronary artery ligation. Females were used as they display significantly lower peri-operative mortality versus males, and also to allow a direct comparison with our previous work relating to the cardioprotective actions of exendin-4 post-MI [18, 27]. Similarly, MI or sham-operated mice were randomly assigned for chronic infusion with either GLP-1(9–36) or saline control, using the same concentration (25 nmol/kg/day; GL Biochem, average purity 90 %) as used in our previous GLP-1 studies [18, 28], via an osmotic minipump (Alzet model 1004) implanted immediately after MI/sham surgery. After 4 weeks, animals were either terminally anaesthetised (2 % isofluorane/oxygen) and hearts arrested in diastole by injection of 10 % KCl, excised and fixed in 10 % neutral-buffered formalin solution, or sacrificed by sodium pentobarbitone overdose (200 mg/kg i.p.) prior to excision of hearts which were frozen in liquid nitrogen and stored at −80 °C for further analyses.

### Assessment of plasma glucose

Terminal blood samples were collected by cardiac puncture into heparinised tubes and centrifuged at 10,000*g* for 10 min to obtain plasma fractions which were analysed for glucose using an enzymatic assay kit (GMRD-002A using glucose oxidase, Analox Ltd) and detected on a GM7 Micro-Stat Analyser (Analox Instruments Ltd).

### Infarct size

Excised hearts were perfused retrogradely with Evans blue dye (1 % in saline) in order to determine area at risk prior to slicing into five serial transverse sections (1 mm) which were incubated in 1 % triphenyltetrazolium chloride at 37 °C to identify infarcted myocardium. Infarct area, area at risk and total left ventricular (LV) area from each section were measured using computerised planimetry (ImageJ), and totalled for all sections. Infarct size was expressed as a percentage of area at risk.

### Echocardiography

Mice were anaesthetised with 1.5 % isofluorane/oxygen, placed on a warming pad, and imaged in the supine position using a Vevo770 ultrasound system with high-frequency 45 MHz RMV707B scanhead (VisualSonics). M-mode parasternal short-axis scans at papillary muscle level were used to quantify interventricular septal thickness in diastole (IVSD), and left ventricular end-diastolic (LVEDD) and end-systolic diameters (LVESD), from which  % fractional shortening was calculated using the equation (LVEDD−LVESD)/LVEDD*100. Parasternal long-axis scans were used to provide additional data on LV end-diastolic (LVEDV) and end-systolic volumes (LVESV) and ejection fraction, whilst pulse-wave Doppler was used to assess mitral valve flow (E/A ratio) and E wave deceleration rate, as reliable measures of diastolic function. All images were analysed by the same observer in a blinded manner in order to minimise variability and bias.

### Cardiac morphometry and in vitro cardiomyocyte studies

Following sacrifice, whole and sectioned hearts were weighed and indexed to total body weight, as a surrogate measure of cardiac hypertrophy. To investigate direct effects of GLP-1(9–36) on in vitro cardiomyocyte hypertrophy, complementary studies were conducted in rat ventricular H9c2 cardiomyoblasts maintained in DMEM containing 10 % FCS, 100 U/ml penicillin and 100 µg/ml streptomycin. At passage, they were plated, cultured to ~50 % confluency and serum-starved for 24 h prior to incubation with phenylephrine (1 μmol/L for 96 h) to induce hypertrophy in the presence or absence of GLP-1(9–36) (0.1 μmol/L) [18]. Cell cross-sectional area was quantified by blinded digital image analysis (NIS-Elements) as an index of hypertrophy.

### Histology and immunohistochemistry

All histological analyses were performed on fixed paraffin-embedded LV sections (5 μm). Cardiac interstitial fibrosis was assessed by picrosirius red staining (0.1 % w/v), excluding coronary vessels and perivascular regions. Data were quantified by digital image analysis (NIS-Elements, Nikon) with the observer blinded to sample identity. Immunocytochemistry for CD45 and F4/80 was performed using rat polyclonal (553076, 1:200; BD Bioscience) and rat monoclonal (MCA497GA, 1:200; AbD Serotec) antibodies, respectively, followed by secondary rabbit anti-rat IgG (P0450, 1:100; Dako) staining, using diaminobenzidine as the chromogen and nuclear counterstaining with haematoxylin.

### Real-time RT-PCR

Total RNA was extracted from LV homogenate or cells using TRI reagent (Sigma-Aldrich), and cDNA synthesised by reverse transcription (Applied Biosystems). mRNA expression was then analysed by reverse transcription polymerase chain reaction (RT-PCR) using fluorescent SYBR Green (Prism 7300, Applied Biosystems) with the following primers (5′–3′): procollagen IαI, forward CCTCAGGGTATTGCTGGACAAC, reverse TTGATCCAGAAGGACCTTGTTTG; fibronectin, forward CCGGTGGCTGTCAGTCAGA, reverse CCGTTCCCACTGCTGATTTATC; CTGF, forward GCTGCCTACCGACTGGAAGAC, reverse GAACAGGCGCTCCACTCTG; MMP-2, forward GACAAGTTCTGGAGATACAATGAAGTG, reverse, CAGGTTATCAGGGATGGCATTC; MMP-9, forward GTGATCCCCACTTACTATGGAAACTC, reverse, GTGCTACACCAAGGCGTGC; TIMP-2, forward GATTCAGTATGAGATCAAGCAGATAAAGA, reverse, GCGAGACCCCGCACACT; IL-1β, forward TGTGGCTGTGGAGAAGCTGT, reverse CAGCTCATATGGGTCCGAGA; IL-6, forward CACGGCCTTCCCTACTTCAC, reverse TGCAAGTGCATCATCGTTGT; MCP-1, forward ATGCTTCTGGGCCTGCTG, reverse GGTGATCCTCTTGTAGCTCTCC; TNF-α, forward, ACTCAACAAACTGCCCTTCTGAG, reverse TTACAGCTGGTTTCGATCCATTT; *Arg1*, forward TTATCGGAGCGCCTTTCTCAA, reverse TGGTCTCTCACGTCATACTCTGT; Fizz1, forward TCCCAGTGAATACTGATGAGA, reverse CCACTCTGGATCTCCCAAGA; IL-10, forward TGCAGGACTTTAAGGGTTACTTGG, reverse GGCCTTGTAGACACCTTGGTC; TGF-β_3_, forward GGAGAGAGTCCAACTGGGTCTG, reverse ACATTTTCCAGTATGTCTCCATTGG. β-actin (primer sequence 5′–3′: forward CGTGAAAAGATGACCCAGATCA, reverse TGGTACGACCAGAGGCATACAG) or GAPDH (primer sequence 5′–3′: forward ACTTTGTCAAGCTCATTTCC, reverse GCAGCGAACTTTATTGATG) was used for normalisation by the comparative Ct method [29].

### RAW264.7 macrophage culture

RAW264.7 murine macrophages (ATCC^®^ TIB-71™) were maintained in DMEM containing 10 % FCS, 100 U/ml penicillin and 100 µg/ml streptomycin. At passage, cells were split into six-well plates, each containing ~1 million cells, prior to incubation in the presence or absence of exendin-4 or GLP-1(9–36) (10 nmol/L) for 4 h. RNA and cDNA were then prepared for real-time RT-PCR mRNA expression analysis of IL-1β, TNF-α, Arg1, Fizz1, IL-10, IL-12, IL-6 and TGF-β_3_ using fluorescent SYBR Green and GAPDH for normalisation.

### Statistical analysis

Data are expressed as mean ± SEM and were analysed by either an unpaired *t* test or one-way ANOVA followed by a Bonferroni’s multiple comparison test, using GraphPad Prism software. P < 0.05 was considered to be statistically significant.

## Results

### Metabolic data

Body weight was not different between groups (sham control: 21.4 ± 0.4, MI control: 21.5 ± 0.2, sham GLP-1(9–36): 21.6 ± 0.5, MI GLP-1(9–36): 21.0 ± 0.3 g; *n* = 8, P = NS). Similarly, chronic infusion with GLP-1(9–36) had no effect on plasma glucose (MI control: 8.7 ± 0.2, MI GLP-1(9–36): 9.2 ± 0.2 mmol/L; *n* = 8–9, P = NS).

### Infarct size

**Fig. 1:**
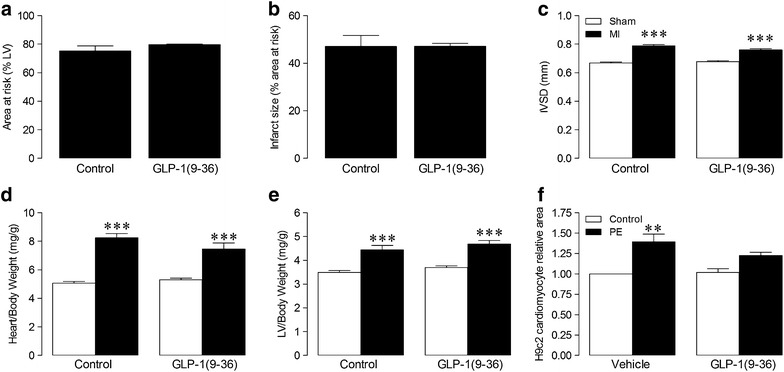
Effect of GLP-1(9–36) on infarct size and cardiomyocyte remodelling post-MI. **a** area at risk and **b** infarct size assessed 4 weeks following MI (*n* = 5–6). **c** Interventricular septal thickness in diastole (IVSD), assessed by echocardiography (*n* = 11–19). **d–e** cardiac morphometry normalised to body weight (*n* = 11–17). **f** phenylephrine-induced hypertrophy in H9c2 cardiomyoblasts (*n* = 4). *White columns*, sham (**c–e**) or untreated cells (**f**); *black columns*, MI (**a**–**e**) or phenylephrine-treated cells (**f**); mean ± SEM. ^**^P < 0.01, ^***^P < 0.001 versus corresponding sham/untreated control

Importantly, both area at risk (control: 75.3 ± 3.4, GLP-1(9–36): 79.6 ± 0.4 %LV; n ≥ 5) and infarct size (control: 47.1 ± 4.6, exendin-4: 47.2 ± 1.2 %LV; n ≥ 5) were comparable between MI groups at 4 weeks (Fig. 1a–b), thereby facilitating specific investigation of direct effects on chronic remodelling. Similarly, survival at 4 weeks post-MI was not different between groups (MI control: 90.0 %, *n* = 30; MI GLP-1(9–36): 87.0 %, *n* = 23; P = NS). No deaths were observed in the sham groups (*n* = 36).

### Cardiomyocyte hypertrophy

Chronic infusion with GLP-1(9–36) had no effect on MI-induced myocardial hypertrophy, as assessed by echocardiographic IVSD (Fig. 1c) and cardiac morphometry (whole heart and LV/body weight ratio, Fig. 1d–e), although in vitro phenylephrine-induced hypertrophy of H9c2 cardiomyoblasts was reduced by GLP-1(9–36) (Fig. 1f).

### Cardiac function

**Fig. 2:**
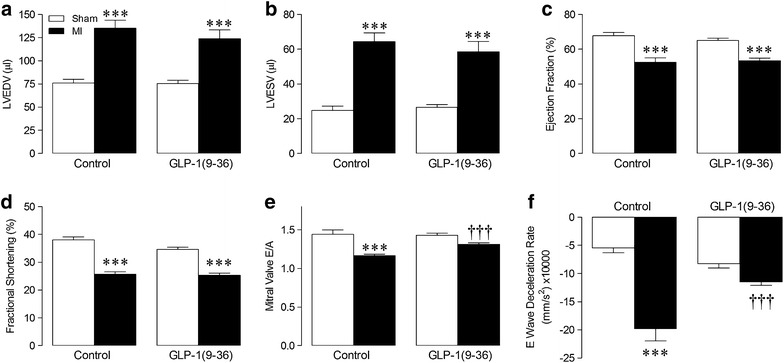
Effect of GLP-1(9–36) on cardiac function post-MI. **a** LV end-diastolic volume (LVEDV), **b** LV end-systolic volume (LVESV), **c** LV ejection fraction, **d** LV fractional shortening, **e** mitral valve E/A ratio, and **f** E wave deceleration rate, assessed by echocardiography (*n* = 8–19). *White columns*, sham; *black columns*, *MI* mean ± SEM. ^***^P < 0.001 versus corresponding sham. ^†††^P < 0.001 versus corresponding MI

Echocardiography data are presented in Fig. 2. Importantly, heart rate remained similar between all groups (sham control: 454 ± 15, MI control: 493 ± 15, sham GLP-1(9–36): 453 ± 0.10, MI GLP-1(9–36): 485 ± 12 bpm; *n* = 8–19, P = NS). LV diastolic and systolic chamber size, as measured by LVEDV and LVESV, respectively, which were both increased after MI, were unaltered by GLP-1(9–36), whilst LV systolic dysfunction post-MI, indicated by reduced ejection fraction and fractional shortening, was also unaffected (Fig. 2a–d). However, decreased mitral valve E/A ratio and increased E wave deceleration rate post-MI, which are reliable indicators of diastolic dysfunction (reflecting reduced LV filling and compliance, respectively) were both attenuated by GLP-1(9–36) treatment (Fig. 2e–f).

### Extracellular matrix remodelling

**Fig. 3:**
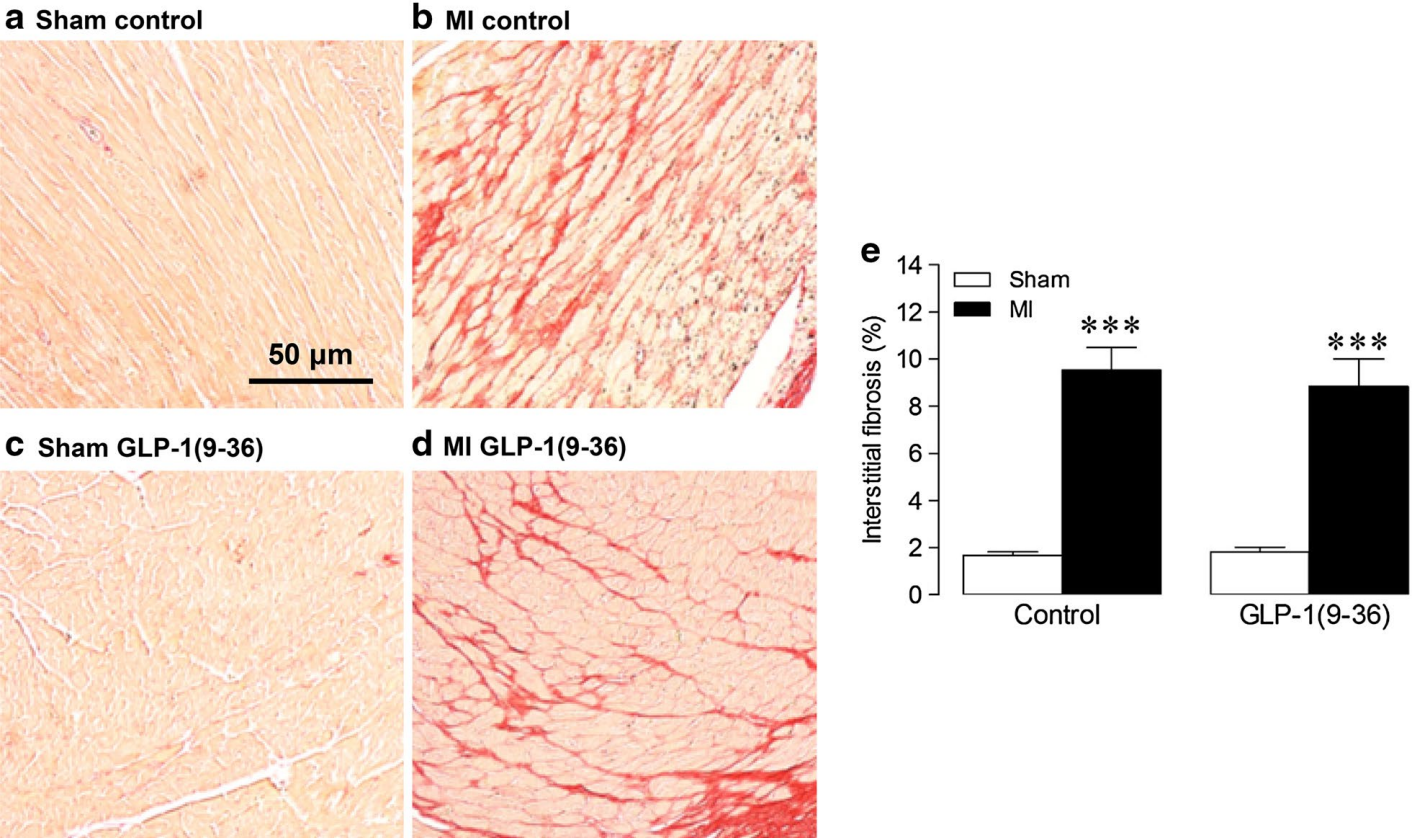
Effect of GLP-1(9–36) on interstitial fibrosis post-MI. **a**–**d** Representative LV sections stained with picrosirius red to assess interstitial fibrosis, with **e** quantification data (*n* = 5–6). *White columns,* sham; *black columns*, *MI* mean ± SEM. ^***^P < 0.001 versus corresponding sham

**Fig. 4:**
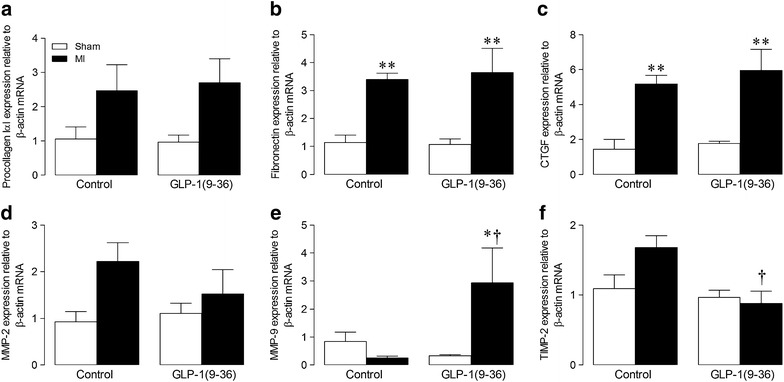
Effect of GLP-1(9–36) on extracellular matrix gene expression post-MI. LV mRNA expression of **a** procollagen IαI, **b** fibronectin, **c** CTGF, **d** MMP-2, **e** MMP-9, and **f** TIMP2 by real-time RT-PCR (*n* = 5-6). *White columns*, sham, *black columns*, *MI* mean ± SEM. ^*^P < 0.05, ^**^P < 0.01 versus corresponding sham; ^†^P < 0.05 versus corresponding MI

Interstitial fibrosis, assessed by picrosirius red staining, was markedly increased 4 weeks post-MI but was unaltered by GLP-1(9–36) (Fig. 3). Similarly, chronic treatment with GLP-1(9–36) had no effect on MI-induced mRNA expression of key pro-fibrotic genes, procollagen IαI, fibronectin and CTGF (Fig. 4a–c). However, GLP-1(9–36) treatment induced MMP-9 mRNA expression and attenuated increased TIMP-2 mRNA expression post-MI, and also tended to reduce MMP-2 mRNA (Fig. 4d–f).

### Myocardial inflammation

**Fig. 5:**
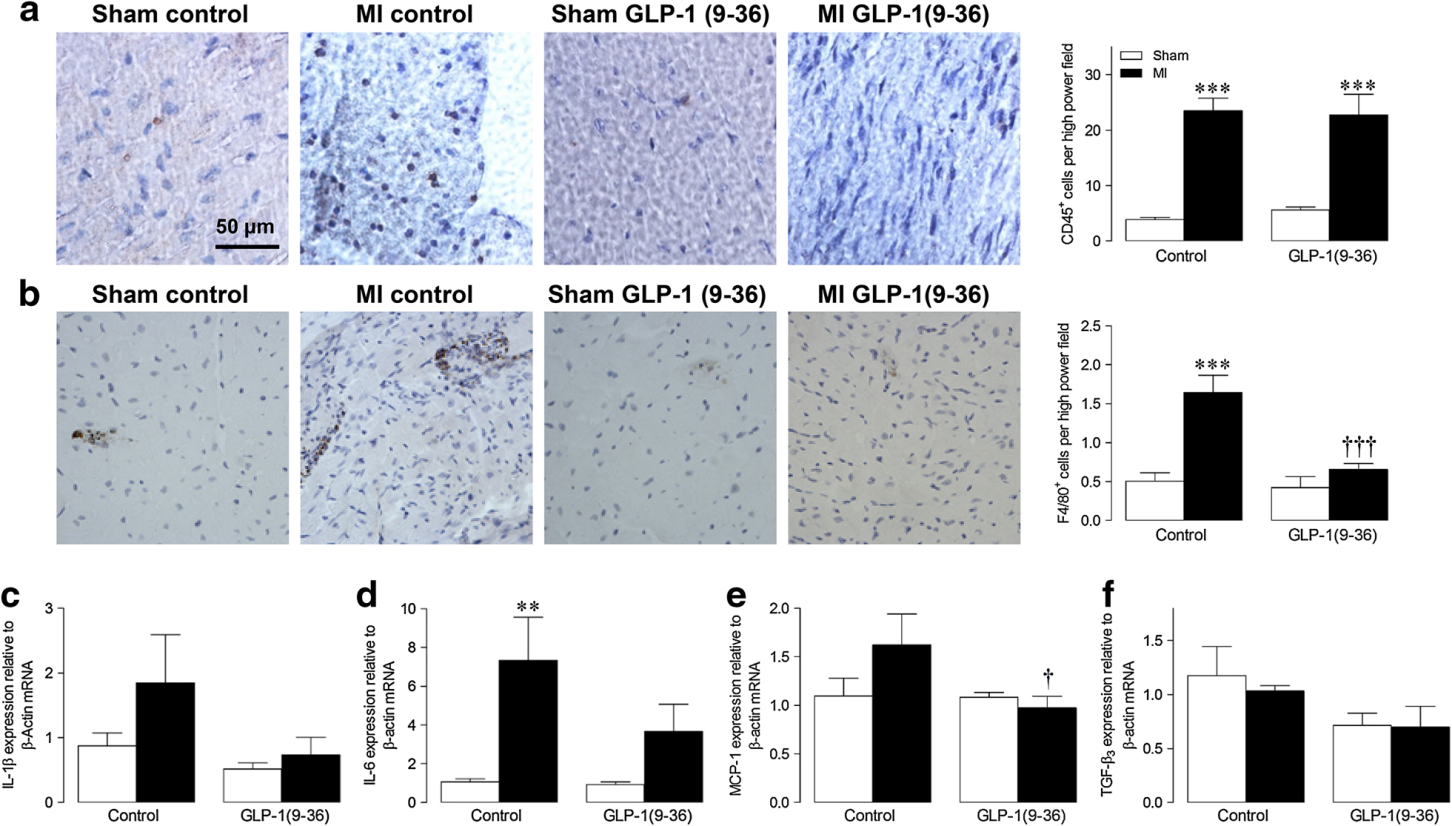
Effect of GLP-1(9–36) on myocardial inflammation post-MI. Representative LV sections stained for **a** CD45 and **b** F4/80, together with mean quantification data by analysis of 10 high power fields per section (n = 5–6). LV mRNA expression of cytokines **c** IL-1β, **d** IL-6, **e** MCP-1, and **f** TGF-β_3_ by real-time RT-PCR (n = 5–6). *White columns*, sham, *black columns*, *MI* mean ± SEM. ^**^P < 0.01, ^***^P < 0.001 versus corresponding sham; ^†^P < 0.05, ^†††^P < 0.001 versus corresponding MI

Cardiac infiltration of both CD45^+^ and F4/80^+^ cells was increased by approximately 5-fold and threefold, respectively, in MI versus sham animals (Fig. 5a–b). Whilst numbers of CD45^+^ leukocytes were comparable between control and GLP-1(9–36)-treated MI hearts, MI-induced infiltration of F4/80^+^ macrophages were markedly reduced by GLP-1(9–36). Similarly, increased mRNA expression of pro-inflammatory IL-1β, IL-6 and MCP-1 in MI hearts was reduced by GLP-1(9–36), although mRNA expression of the key pro-fibrotic cytokine, TGF-β_3_, was unaltered between groups (Fig. 5c–f).

### Cell studies

**Fig. 6:**
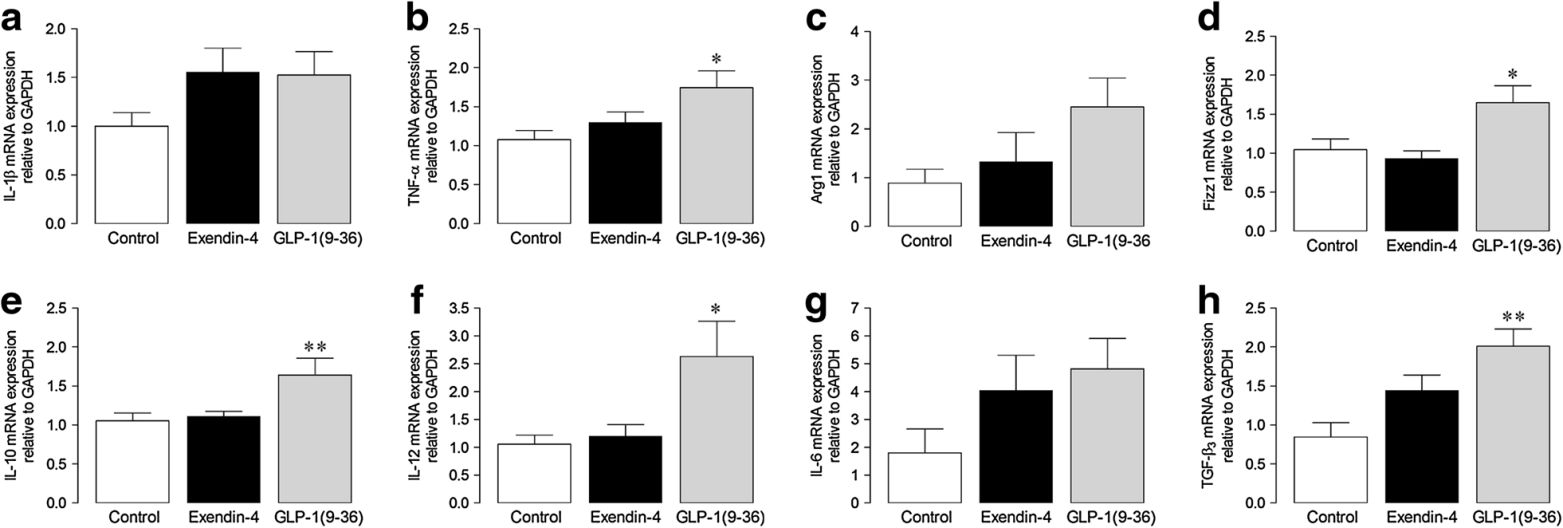
Effect of GLP-1(9–36) on inflammatory gene expression in RAW264.7 macrophages. mRNA expression of **a** IL-1β, **b** TNF-α, **c** Arg1, **d** Fizz1, **e** IL-10, **f** IL-12, **g** IL-6, and **h** TGF-β_3_ by real-time RT-PCR (*n* = 5–12). Mean ± SEM. ^*^P < 0.05, ^**^P < 0.01 versus corresponding control

In order to investigate direct inflammatory effects of GLP-1(9–36), complementary studies were conducted in RAW264.7 macrophages (Fig. 6). Notably, treatment of these cells for 24 h with 10 nmol/L GLP-1(9–36) resulted in a general increase in mRNA expression of several macrophage response genes, associated with both an M1 (IL-1β, TNF-α, Arg1, IL-12, IL-6) and M2 (Fizz1, IL-10, TGF-β_3_) cell phenotype, compared with cells incubated with the same concentration of exendin-4.

## Discussion

In this study, we clearly demonstrate that metabolically-inactive GLP-1(9–36) exerts selective actions on experimental post-MI remodelling, a finding which has obvious clinical significance. Specifically, we have shown that GLP-1(9–36) is protective against the development of post-MI diastolic dysfunction and modulates myocardial inflammation, but appears to have limited effects on ECM and cardiomyocyte remodelling in this setting. Complementary cell studies found that acute GLP-1(9–36) treatment potentiated basal macrophage response gene expression to a greater extent than exendin-4, indicating that this often overlooked GLP-1 breakdown product may exert important differential actions on infiltrating inflammatory cells, which may thereby influence post-MI remodelling.

### Choice of experimental model for specific study of GLP-1(9–36) in chronic post-MI remodelling

The rationale for this study was based on the fact that GLP-1(9–36) has been reported to mediate several important cardiovascular actions in experimental models, including promotion of vascular relaxation, modulation of cardiac function, and protection against ischemia–reperfusion injury [4, 5, 24]. As we have recently shown that the stable GLP-1 mimetic, exendin-4, exerts selective protection against chronic post-MI remodelling, and both GLP-1R activation and GLP-1(9–36) reduce infarct size in response to acute ischaemia [18, 24], it seemed logical to investigate whether GLP-1(9–36) may also mediate specific actions on the remote myocardium after chronic ischaemia. This may be particularly relevant to the clinical setting given the increasing interest in the cardiovascular actions of established GLP-1 therapies in diabetic patients with heart failure [1, 25, 26], particularly DPP-4 inhibitors, which inhibit the breakdown of native GLP-1(7–36)amide, thereby significantly reducing circulating levels of its breakdown product, GLP-1(9–36). In this regard, it is important to note that our chosen experimental approach employing continuous infusion of GLP-1(9–36) commencing immediately after MI or sham surgery, as used in our previous study [18], facilitated specific investigation of direct effects on chronic post-MI remodelling in the absence of indirect effects further to reported benefits on acute infarct remodelling in response to GLP-1 treatment [10, 11, 30]. Indeed, area at risk and infarct size assessed at 4 weeks post-MI were similar between control and GLP-(9–36)-treated mice indicating that the observed chronic ventricular remodelling changes were likely to be due to direct cardiac actions of the peptide. Furthermore, as expected, metabolically inactive GLP-1(9–36) had no effect on either body weight or plasma glucose.

### GLP-1(9–36) has minimal actions on cardiomyocyte remodelling

In contrast to exendin-4, which we recently reported to exert modest protection against post-MI cardiomyocyte remodelling [18], GLP-1(9–36) treatment in MI mice had no effect on cardiac morphology, as assessed by echocardiographic IVSD and post-mortem heart and LV mass, although a small reduction in in vitro phenylephrine-induced H9c2 cardiomyoblast hypertrophy was observed. Indeed, although several studies have shown that GLP-1 is protective against acute cardiomyocyte remodelling [5, 23, 31], it appears that GLP-1 may have limited direct effects on this cell type in vivo [1, 18]. However, that is not to say that indirect modulation of cardiomyocyte autocrine or paracrine signalling by GLP-1 peptides should be discounted. As far as we are aware, the effects of GLP-1(9–36) on in vivo cardiomyocyte remodelling have not been previously investigated, although several groups have reported beneficial actions of GLP-1(9–36) on cardiac contractile function. For example, GLP-1(9–36) was shown to reduce infarct size and improve functional recovery after ischaemia–reperfusion in isolated mouse hearts, whilst the beneficial effects of GLP-1R activation in this setting persisted in the absence of a functional GLP-1R or in the presence of the GLP-1R antagonist, exendin(9–39), and were abolished by the DPP-4 inhibitor, sitagliptin [5, 23], strongly suggesting that these effects are mediated by GLP-1(9–36). Indeed, selective disruption of the cardiomyocyte GLP-1R demonstrated that it was not required for GLP-1 agonist-induced cardioprotection [32], further highlighting a potential role for GLP-1(9–36). Similarly, continuous infusion of both native GLP-1(7–36)amide and GLP-1(9–36) improved systolic/diastolic function and myocardial metabolism in dogs with rapid pacing-induced dilated cardiomyopathy, although GLP-1(9–36) failed to increase cardiac output during regional myocardial ischaemia in pigs [12, 33].

### GLP-1(9–36) specifically protects against diastolic dysfunction post-MI

Here, we found that whilst GLP-1(9–36) had no effect on echocardiographic systolic function post-MI, as indexed by LVESV, ejection fraction and fractional shortening, GLP-1(9–36) significantly attenuated diastolic dysfunction, as indicated by increased mitral valve E/A ratio (improved LV filling) and reduced E wave deceleration rate (improved LV compliance), although LV dilatation associated with MI remained unchanged. It therefore appears that GLP-1(9–36) promotes preservation of cardiac function post-MI, although it should be noted that the extent of this effect may be less pronounced than that observed with exendin-4 in the same model [18]. Diastolic function is known to be particularly sensitive to ECM alterations so given the improvement in mitral valve flow in GLP-1(9–36)-treated mice, it would seem logical to expect parallel reduction of cardiac fibrosis. However, interstitial fibrosis in MI mice was not affected by GLP-1(9–36). Similarly, pro-fibrotic gene expression was largely unaltered in hearts from GLP-1(9–36)-treated animals, although mRNA expression of TIMP2 was reduced in MI GLP-1(9–36) mice in parallel with a tendency towards reduced expression of its main target, MMP-2, a major driving force for ECM remodelling which is upregulated in MI [34]. Indeed, MMP-9, which is typically thought to promote adverse ECM remodelling [35], was oppositely regulated after MI in the GLP-1(9–36) treated animals compared to control, thereby highlighting differential actions on the ECM which may underlie the observed attenuation of diastolic dysfunction. Taken together, these data would appear to suggest that GLP-1(9–36) may influence ECM turnover rather than cardiac fibrosis per se, thereby resulting in improved LV relaxation, compliance, and diastolic function, although a more robust analysis of ECM signalling is clearly required before any definite conclusions can be drawn.

### GLP-1(9–36) reduces myocardial inflammation via specific effects on infiltrating macrophages

It is becoming increasingly evident that myocardial inflammation represents a key regulator of post-MI remodelling, which directs specific actions on individual components in this setting, but which is thought to preferentially target the ECM [36]. Indeed, we have previously reported that the GLP-1 mimetic, exendin-4, protects against adverse cardiac remodelling in both normoglycaemic and diabetic mice via specific actions on infiltrating inflammatory cells [18, 37]. In this regard, whilst GLP-1(9–36) did not affect the marked myocardial infiltration of CD45^+^ leukocytes associated with MI, increased numbers of F4/80^+^ macrophages, which are particularly prevalent in the ischaemic heart [18, 38], were normalised by GLP-1(9–36). This was associated with reduced expression of the pro-inflammatory cytokines, IL-1β, IL-6 and MCP-1, which are critical for fibroblast differentiation, although mRNA levels of TGF-β_3_ which also upregulates MMP/TIMP expression, were unaltered [34, 39]. Interestingly, complementary in vitro experiments performed using RAW264.7 murine macrophages revealed a clear potentiation of basal mRNA expression of macrophage response genes, which are known to regulate tissue remodelling and repair, in the presence of GLP-1(9–36) compared with exendin-4, which we have previously reported to modulate resident and secreted cytokine expression in bone marrow-derived cells under both normal and high glucose conditions [18, 37, 40, 41]. Importantly, GLP-1(9–36) appeared to upregulate both pro-inflammatory (IL-1β, TNF-α, IL-6, IL-12, Arg1) and tissue protective cytokines (IL-10, Fizz1), in addition to TGF-β_3_ which is a major driver of ECM remodelling [38, 39], highlighting global activation of macrophage function. It should be noted that whilst the employed concentration of GLP-1(9–36) in these in vitro experiments was based on that used in previous studies, it is higher than circulating levels typically found in vivo [24, 42, 43]. Nonetheless, taken together with the observation that MI-induced macrophage infiltration was almost completely prevented by GLP-1(9–36), these data strongly suggest that this purportedly inactive peptide exerts significant actions which are relevant to cardiac remodelling. Whilst these findings are consistent with a previous report of GLP-1(9–36)-mediated inhibition of chemokine-induced migration of human CD4-positive lymphocytes [44], which is established as an early and critical step in atherogenesis, ours is the first study to specifically link an immunomodulatory effect of GLP-1(9–36) to cardioprotection. Although this apparent modulation of the myocardial inflammatory response by GLP-1(9–36) is most likely to impact upon ECM remodelling and thereby explain the observed preservation of cardiac function, it is also possible that reduced cardiac macrophage infiltration and associated changes in gene expression may also influence cardiomyocyte function due to modulation of established paracrine communication between these two cell populations [45]. Interestingly, recent reports that increased mitochondrial ROS generation associated with both high glucose exposure in human endothelial cells and Alzheimer’s disease in mouse hippocampal tissue, is attenuated by GLP-1(9–36) [21, 46, 47], suggest that such antioxidant actions of GLP-1(9–36) may underlie the observed attenuation of post-MI remodelling, specific components of which are known to be significantly influenced by ROS [48].

## Conclusion

Although, the precise role of GLP-1(9–36) in the ischaemic heart remains unclear and the mechanisms underlying its apparent protective actions require detailed further investigation, it is evident that such effects are likely to be clinically significant so should be considered in the context of GLP-1 therapy in patients with cardiovascular disease. Indeed, it is possible that the apparently disappointing results of the first large-scale cardiovascular trials of the DPP-4 inhibitors saxagliptin and alogliptin, which are likely to markedly reduce circulating levels of GLP-1(9–36), could be at least partly explained by the absence of this endogenous GLP-1 breakdown product. In support of this supposition, a recent experimental study reported that administration of a preclinical DPP-4 inhibitor, MK-0626, to high fat-fed diabetic mice resulted in increased cardiac fibrosis and impaired ventricular function [49]. Together with our previous findings with exendin-4 in the setting of both experimental MI and diabetes, the data presented here suggest that selective targeting of specific remodelling pathways, with consideration of both GLP-1R activation and GLP-1 breakdown, may overcome the known pleiotropic nature of GLP-1 signalling. Such novel approaches utilising, for example, specific GLP-1 peptide modification strategies [50], may thereby improve the effectiveness of GLP-1-based strategies for cardiovascular disease.

## Abbreviations

DPP-4: dipeptidyl peptidase-4
IVSD: interventricular septal thickness in diastole
ECM: extracellular matrix
GLP-1: glucagon-like-peptide-1
GLP-1R: glucagon-like-peptide-1 receptor
LV: left ventricle
LVEDD: left ventricular end-diastolic diameter
LVEDV: left ventricular end-diastolic volume
LVESD: left ventricular end-systolic diameter
LVESV: left ventricular end-systolic volume
MI: myocardial infarction
RT-PCR: reverse transcription polymerase chain reaction
ROS: reactive oxygen species
